# Supplementary material for: Field-linear anomalous Hall effect and Berry curvature induced by spin chirality in the kagome antiferromagnet Mn3Sn
Source: Nat Commun. 2023 Mar 24;14:1642. doi: 10.1038/s41467-023-37076-w (PMC10039076; doi:10.1038/s41467-023-37076-w)
Supplement: Supplementary file 1 — Supplementary Information [file 41467_2023_37076_MOESM1_ESM.pdf]

Supplementary Information for "Field-linear anomalous Hall effect and Berry curvature induced by spin chirality in the kagome antiferromagnet Mn_3Sn"

SUPPLEMENTARY NOTES

Supplementary Note 1: Hall and Nernst conductivity

The intrinsic transport responses are different conductivity tensor, such as the electric conductivity tensor σ, the thermoelectric conductivity tensor α, and the thermal conductivity tensor κ. These three conductivity tensors are interrelated by the Onsager relations, as seen in the formula S1 and S2. Here J and J_Q are electric and heat current respectively. In presence of a magnetic field, their off-diagonal components will appear, the σ_{xy} represents the Hall conductivity, the α_{xy} represents the Nernst conductivity, and the κ_{xy} represents the thermal Hall conductivity [1].

$$J = \sigma \cdot E - \alpha \cdot \nabla T \tag{S1}$$

$$J_Q = \alpha T \cdot E - \kappa \cdot \nabla T \tag{S2}$$

To quantify these off-diagonal conductivity components, such as the field induced anomalous Hall (σ_{xy}^{FA}) and Nernst (α_{xy}^{FA}) conductivity studied in this work, as seen in Supplementary Figure 1e and f, we need to measured different longitudinal and transverse electric and thermoelectric coefficients, such as the resistivity (ρ_{xx}), the Hall resistivity (ρ_{xy}), the Seebeck coefficient (S_{xx}) and the Nernst coefficient (S_{xy}), as seen in Supplementary Figure 1a-d. S3 and S4 show the conversion formula deduced from S1 and S2 and considering $\rho_{xx} = \rho_{yy}, S_{xx} = S_{yy}$.

$$\sigma_{xy} \approx \frac{-\rho_{xy}}{\rho_{xx}^2} \tag{S3}$$

$$\alpha_{xy} \approx \frac{\rho_{xx} S_{xy} - \rho_{xy} S_{xx}}{\rho_{xx}^2} \tag{S4}$$

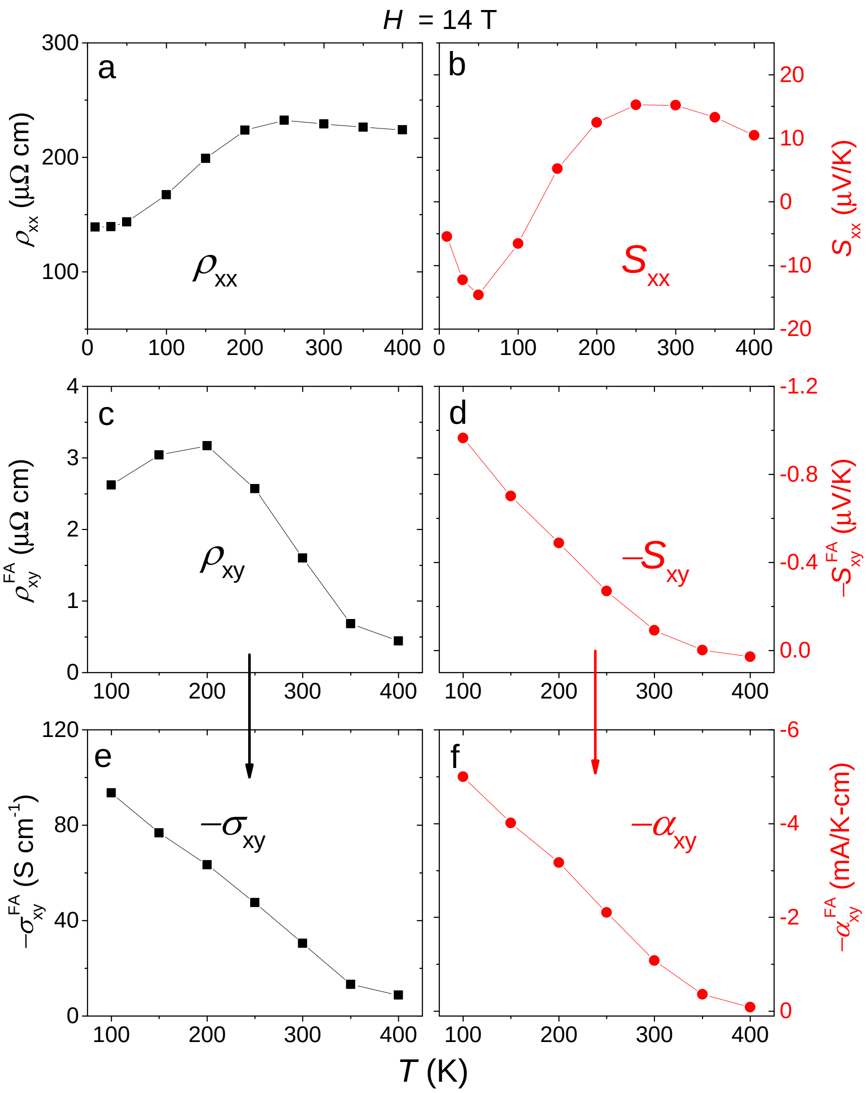

Supplementary Figure 1: Temperature dependent electric and thermoelectric transport responses. (a-b) The in-plane resistivity (ρ_{xx}) and Seebeck coefficient (S_{xx}) at 14 T with temperature varying from 400 K to 10 K. (c-d) The field induced anomalous Hall resistivity (ρ_{xy}^{FA}) and Nernst coefficient (S_{xy}^{FA}) at 14 T with temperature varying from 400 K to 100 K.(e-f) The field induced anomalous Hall conductivity (σ_{xy}^{FA}) and Nernst conductivity (α_{xy}^{FA}) calculated from data in (a-d).

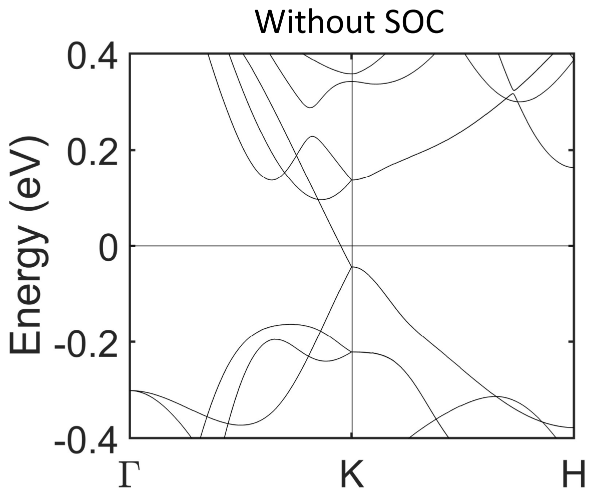

Supplementary Figure 2: The Weyl nodal line along $K-H$ without and with SOC in the absence of spin canting. The spin-structure remains same for both calculations. Without SOC, the K-H line is always doubly-degenerated which forms a Weyl nodal line. Then, the SOC weakly lifts the degeneracy and induced tiny gaps along the $K-H$ line. It excludes that the seemingly band crossing at K is a Weyl point.

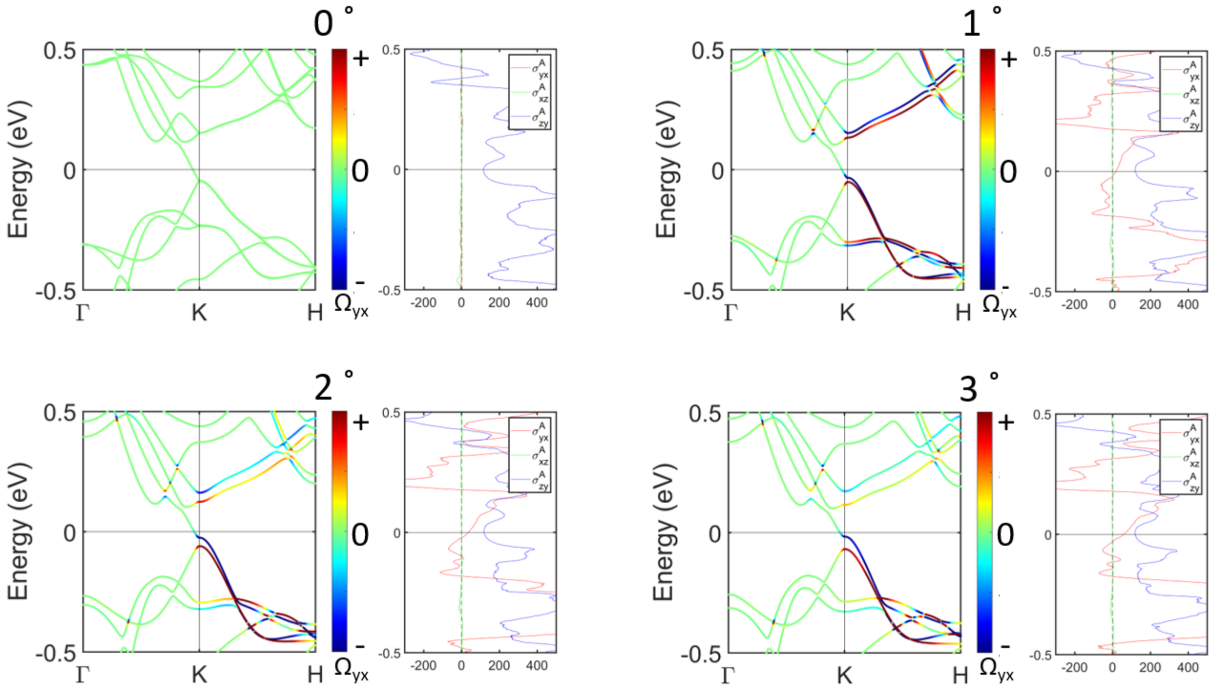

Supplementary Figure 3: Canting angle dependent band structure with Ω_{yx} and σ_{yx}. Results with six different canting angle varying from 0 ° to 3 ° are shown. With in-plane spin structure (0 °) the Ω_{xy} is exactly zero, because of the mirror symmetry. Induced spin canting starts to significantly gap out the Weyl nodal line and generate the Berry curvature Ω_{xy} on the Fermi surface. Increasing the canting angle enlarges the band splitting. As the consequence, the Ω_{xy} is smeared out and reaches the Fermi surface.

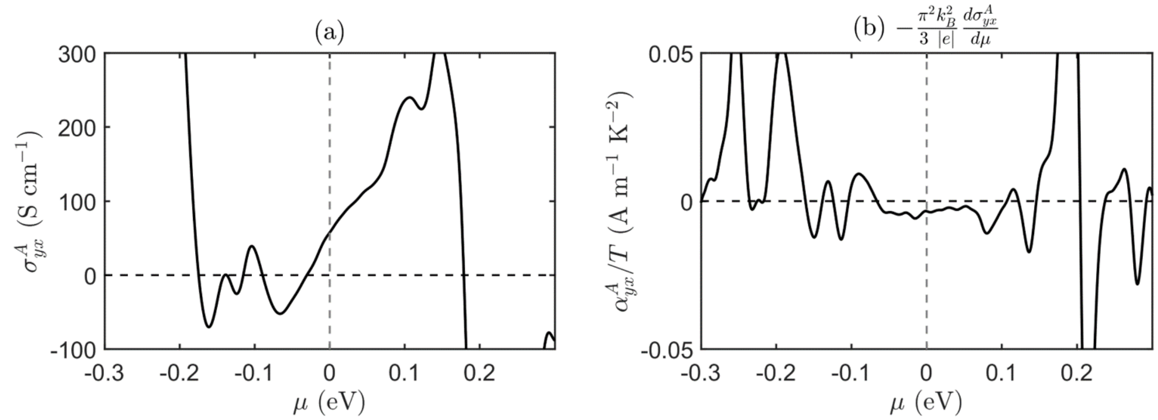

Supplementary Figure 4: Calculated anomalous Nerst coefficient α_{yx} via the Mott's relation for the 3° spin canting. (a) Anomalous Hall conductivity at 0 K as varying the chemical potential μ. The charge neutral point is set to zero. (b) The value of α^A_{yx}/T.

SUPPLEMENTARY REFERENCES

[1] Li, X. *et al.* Anomalous nernst and righi-leduc effects in mn_3sn: Berry curvature and entropy flow. *Phys. Rev. Lett.* **119**, 056601 (2017). URL https://journals.aps.org/prl/abstract/10.1103/PhysRevLett.119.056601.
